# Occult Serious Bacterial Infections in Neonates and Infants Up to Three Months of Age with Bronchiolitis: Are Invasive Cultures Required?

**DOI:** 10.3390/antibiotics13080702

**Published:** 2024-07-26

**Authors:** Domenico Umberto De Rose, Venere Cortazzo, Marilena Agosta, Paola Bernaschi, Maria Paola Ronchetti, Velia Chiara Di Maio, Alessandra Di Pede, Jole Rechichi, Annabella Braguglia, Carlo Federico Perno, Andrea Dotta

**Affiliations:** 1 Neonatal Intensive Care Unit, “Bambino Gesù” Children’s Hospital IRCCS, 00165 Rome, Italy; domenico.derose@opbg.net (D.U.D.R.); mariapaola.ronchetti@opbg.net (M.P.R.); andrea.dotta@opbg.net (A.D.); 2 PhD Course in Microbiology, Immunology, Infectious Diseases, and Transplants (MIMIT), Faculty of Medicine and Surgery, “Tor Vergata” University of Rome, 00133 Rome, Italy; 3 Microbiology and Diagnostic Immunology Unit, “Bambino Gesù” Children’s Hospital IRCCS, 00165 Rome, Italy; venere.cortazzo@opbg.net (V.C.); paola.bernaschi@opbg.net (P.B.); veliachiara.dimaio@opbg.net (V.C.D.M.); carlofederico.perno@opbg.net (C.F.P.); 4 Neonatal Sub-Intensive Care Unit and Follow-Up, “Bambino Gesù” Children’s Hospital IRCCS, 00165 Rome, Italy; alessandra.dipede@opbg.net (A.D.P.); jole.rechichi@opbg.net (J.R.); annabella.braguglia@opbg.net (A.B.)

**Keywords:** sepsis, newborns, RSV, rhinovirus, viruses, influenza, respiratory infections

## Abstract

(1) Background: The literature reports a low risk of serious bacterial infections (SBIs) in febrile infants presenting with bronchiolitis or respiratory syncytial virus infection, but current microbiological techniques have a higher accuracy. (2) Methods: We assessed the risk of SBIs in neonates and infants with bronchiolitis from 2021 to 2023. We also evaluated C-reactive protein, procalcitonin, and leukocyte values. (3) Results: We included 242 infants. Blood cultures (BCs) were performed in 66/242 patients, with a positivity rate of 9.1% (including one BC with *Staphylococcus hominis*, considered as a contaminant). The cerebrospinal fluid culture was performed in 6/242 patients, and the results were all negative. Infection markers did not discriminate infants with positive BCs from those with negative ones. (4) Conclusions: Blood cultures should be performed in neonates and young infants with bronchiolitis fever, as the sepsis risk is not negligible. Conversely, our proposed algorithm is to wait for the respiratory panel results before decision-making for a lumbar puncture. Further studies are needed to understand lumbar puncture requirements.

## 1. Introduction

Bronchiolitis is a common lower respiratory tract infection affecting children younger than 24 months, usually from October to November and from March to April in Europe [1]. However, the risk of a serious bacterial infection (SBI) should always be considered when fever occurs in the first months of life. Laboratory tests such as C-reactive protein (CRP) and procalcitonin (PCT) can significantly improve the identification of children with an SBI when there is a fever without a source [2]; however, their interpretation is difficult when there is overlapping bronchiolitis.

The literature reports that the risk of SBIs in febrile infants presenting with bronchiolitis or respiratory syncytial virus infection is very low [3,4]. However, these studies did not assess the risk of SBIs in the case of high CRP and CPT values in these children. Furthermore, in most cases, these studies often included infants beyond the first months of life, and current microbiological techniques have greater diagnostic accuracy than they did previously [5,6].

Considering these reasons, we conducted a retrospective study to compare the risk of SBIs in neonates and young infants admitted because of bronchiolitis to assess the need for blood culture and cerebrospinal fluid culture.

## 2. Results

From 1 October 2021 to 31 March 2023, we admitted 247 neonates and young infants with acute bronchiolitis. Five infants were excluded because of incomplete data. Therefore, we included 242 infants, of whom 111 were males (45.9%). One hundred fifty-eight (65.3%) were neonates, whereas eighty-four (34.7%) were within three months of life. The median gestational age was 39 weeks (IQR 38–40); the median birthweight was 3200 g (IQR 2870–3473); and the median age at admission was 24 days of life (IQR 16–35). Twenty-four patients (9.9%) were born preterm (range: 25–36 weeks of gestational age). Table 1 shows the viruses causing bronchiolitis in our patients.

At admission, CRP and leukocyte values were available for all infants, whereas PCT was measured in 135 infants (55.8%).

The median CRP value was 0.46 mg/dL (IQR 0.11–1.55), with a maximum value of 19.62 mg/dL; the median PCT value was 0.17 ng/mL (IQR 0.12–0.35), with a maximum value of 92.70 ng/mL; median leukocytes were 10,100/mm^3^ (IQR 8000–12,390), with a maximum value of 23,020/mm^3^.

Invasive cultures (blood cultures and/or cerebrospinal fluid cultures) were performed in 66 patients (27.3%) because of fever in 44/66 infants (66.7%) or abnormal laboratory tests (CRP and/or PCT values) in 21/66 infants (31.8%).

We found significant differences in CRP values between infants with fever and those without fever, whereas we found no significant differences in PCT and leukocyte values (Table 2).

### 2.1. Blood Cultures

Blood cultures were performed in 66/242 patients (27.3%); in 16/66 patients (24.2%), a T2 Bacteria panel was also performed due to fever persistence. A positive blood culture was found in 3/66 patients (4.5%), identifying *Streptococcus pneumoniae*, *Staphylococcus hominis*, and *Staphylococcus aureus* in each case, respectively. A positive T2 Bacteria panel was found in 3/16 patients (18.8%), identifying *Enterococcus faecium*, *Escherichia coli,* and *Pseudomonas aeruginosa*, respectively. Using T2MR, the incidence of positive BC results increased from 3/66 patients (4.5%) to 6/66 patients (9.1%). Five infants with blood cultures positive for pathogens were treated with empirical antibiotics (all patients with positive BCs except the patient with *Staphylococcus hominis*, which was considered a contaminant).

In the group with BCs positive for pathogens, 2/5 infants (40%) were born preterm (26 and 36 weeks) versus 3 term infants: all infants were hospitalized at the emergency department and came from their homes. Conversely, in the group without detected pathogens, 8/61 infants (13.1%) were born preterm (*p* = 0.162).

In Table 3, we report the CRP and PCT values in infants with positive blood cultures.

Infection markers did not discriminate infants with positive BCs from those with negative ones (Table 4).

Indeed, the choice of performing BCs cannot be guided only by infection biomarkers: in Table 5, we report how many infants had positive infection biomarkers in patients without positive BCs results (*n* = 237), including those with negative BCs (*n* = 61) and those in whom BCs were not performed (*n* = 176).

### 2.2. Cerebrospinal Fluid Cultures

Because of fever persistence, we performed lumbar punctures in 6/242 patients (2.5%). The culture and the film-array molecular testing of all cerebrospinal fluid samples tested negative. In these six patients with negative CSF samples, the median CRP value was 1.28 mg/dL (IQR 0.44–1.98; maximum value: 5.18); the median PCT value was 0.56 ng/mL (IQR 0.29–23.23; maximum 90.5); and the median leukocytes were 8445/mm^3^ (IQR 7953–9207; maximum: 11,640).

## 3. Discussion

In this study, we report how the incidence of occult serious bacterial infections in neonates and young infants with bronchiolitis can be higher than previously reported [3,4]. Furthermore, these previous studies did not investigate the risk of a positive invasive culture in the case of high CRP and PCT values, and current microbiological techniques have greater diagnostic accuracy than they did previously [5,6]. To the best of our knowledge, this is the first study to evaluate the infection biomarkers’ results in this cohort.

To date, merging the data from 26 studies from low-income and middle-income countries, the global incidence of sepsis is 2824 sepsis cases per 100,000 live births, with a mortality of 17.6% [7]; the incidence and mortality are lower in high-income countries [8]. However, given the risk of late-onset neonatal sepsis, fever should always be considered an important finding in this age category. Neonatal sepsis can have varied presentations, including hypo- or hyperthermia, irritability or lethargy, apnea or tachypnea, bradycardia or tachycardia, poor feeding, excessive sleepiness, or fussiness [9].

Clinical practice guidelines about evaluating and managing well-appearing febrile infants 8 to 60 days old suggest obtaining a blood culture and performing a lumbar puncture when there is no evident source of infection [10]. The Rochester, Philadelphia, and Boston criteria all recommend a full septic workup in infants under 28 days of age presenting with fever regardless of other risk factors [11].

Conversely, although it is not common for a patient with a viral infection to have a bacterial infection simultaneously, it is difficult to draw “black or white” conclusions when a respiratory infection is clinically evident, and infants are not well-appearing.

The choice of whether to perform invasive cultures or not can be guided not only by the clinical appearance of the baby but also by an accurate anamnesis (for example, prematurity and previous hospitalization). In the first days of life, the risk of late-onset sepsis is higher than that of early-onset sepsis, especially if maternal vaginal swabs were negative and the mother had no intrapartum signs of infection or if the baby received complete intrapartum antibiotic prophylaxis, although there is a rising trend of life-threatening *Escherichia coli* infections [12].

Furthermore, the presence of older siblings is a well-known risk factor for bronchiolitis [13]. In the presence of other family members with overt viral respiratory infections, such as older brothers with colds who attend kindergarten, it is difficult for the newborn/infant to have a different infection than them.

Contrariwise, the case of a newborn managed in an intensive care unit for bronchiolitis who begins to have a fever after 48–72 h is different, because, in this case, the fever may be a sign of a nosocomial infection acquired during the hospital stay [14].

This study has four limitations: first, information from a single center was retrospectively gathered, and records may not always be comprehensive. Second, in our two neonatal units, we hospitalized only babies with the most severe bronchiolitis, who require respiratory support or intravenous fluids because of feeding difficulties. This could have influenced the positivity rate of the BCs, affecting the denominator of bronchiolitis cases on which to calculate the incidence of overlapping SBIs. Third, concerning the risk of meningitis, in our clinical routine, we perform a lumbar puncture in all febrile neonates when the fever source is not evident or high infection biomarkers have been found, whereas we do not usually perform it in febrile infants with bronchiolitis. Given the lack of definitive criteria for this decision, the choice to perform a lumbar puncture in some infants of this cohort was subjectively made by the clinician who evaluated the baby at that moment. Thus, we could evaluate the positivity rate of CSF in six febrile infants only. On the other hand, all other newborns would not have improved and recovered within a few days without specific treatment if they had unidentified meningitis, but they would have died.

Interestingly, our findings are in line with a recent study on a cohort of 693 infants hospitalized in a pediatric intensive care unit because of severe bronchiolitis, with a median age of 47 days. In our cohort, we found positive BC results in 9.1% of cases (including one BC with *Staphylococcus hominis*), whereas Guitart et al. found positive blood cultures in 13.2% of cases (including six BCs with *Staphylococcus epidermidis* and two with *Staphylococcus hominis*, which were considered contaminants). Similarly to our data, none of the infants had positive CSF results [15]. Recently, lumbar puncture in low-risk febrile infants with bronchiolitis has been considered a “thing we do for no reason^TM^”, inspired by the ABIM Foundation’s Choosing Wisely campaign [16].

## 4. Methods

### 4.1. Study Design

We retrospectively collected data (gender, gestational age, birthweight, age at admission, CRP and PCT values at admission, leukocytes at admission, fever) from the medical records of neonates and infants aged <3 months, admitted to the Neonatal Intensive Care Unit and Neonatal Sub-Intensive Care Unit of “Bambino Gesù” Children’s hospital (Rome, Italy) for bronchiolitis from October 2021 to March 2023. We also collected the results of nasopharyngeal swabs for the identification of respiratory viruses at admission and the results of blood and cerebrospinal fluid cultures.

The primary outcome was the risk of SBIs (sepsis and meningitis) in neonates and infants with bronchiolitis.

The secondary outcome was to evaluate CRP values, PCT values, and leukocytes at admission in case of fever or according to the cultures’ results.

### 4.2. Microbiology Testing

All patients enrolled were studied with nasopharyngeal swabs for the identification of respiratory viruses (influenza virus, respiratory syncytial virus, adenovirus, enterovirus, parainfluenza virus, metapneumovirus, bocavirus, rhinovirus, and coronaviruses, including NL63/229E/OC43 and SARS-CoV-2). Etiological agents were detected on nasopharyngeal aspirates/swabs by the multiplex real-time polymerase chain reaction (RT-PCR) “AllplexTM Respiratory Panel Assays” on All-in-One Platform (Seegene, Republic of Korea), as previously described [17], or by the BioFire FilmArray Respiratory 2.1 Panel (BioMérieux Clinical Diagnostics, Salt Lake City, UT, USA).

In case of fever or high CRP or PCT values, blood cultures (BCs) were collected for a sepsis workup. Fever was defined as a rectally obtained temperature ≥ 38 °C, whereas normal values for CRP and PCT were set at 0.5 mg/dL and 0.5 ng/mL from our laboratory, respectively.

Briefly, blood cultures were performed in accordance with hospital practices and international recommendations for pediatric populations. Each BC bottle was inoculated with 1 mL (Bactec Peds Plus/F medium) of whole blood and incubated at 35 °C in a Bactec 9240/70FX BC system (BD Becton Dickinson Diagnostics, Franklin Lakes, NJ, USA) [18].

When fever persisted, whole blood samples were collected for a T2 magnetic resonance test (T2MR) for bacteria (T2 Bacteria panel) (T2 Biosystems, Lexington, MA, USA). T2Bacteria was performed for a direct-from-blood rapid identification and multiplex detection of the ESKAPE bacteria (*Enterococcus faecium*, *Staphylococcus aureus*, *Klebsiella pneumoniae*, *Acinetobacter baumannii*, *Pseudomonas aeruginosa*, and *Escherichia coli*) most commonly involved in bloodstream infections [6].

In some cases with fever persistence, cerebrospinal fluids (CSFs) were analyzed with standard culture and a Film-Array Meningitis and Encephalitis Panel (ME Panel, BioMérieux Clinical Diagnostics, Salt Lake City, UT, USA) for the rapid PCR-based detection in CSF of 14 targets (6 bacteria, 7 viruses, and a yeast).

All the samples were processed according to routine laboratory workflow, and they were managed according to the standardized protocols of the microbiology laboratory.

### 4.3. Ethical Statement

All the procedures of this study comply with the ethical standards of the institutional and national research committee and with the 1964 Helsinki Declaration and its later amendments or comparable ethical standards [19]. Personal data were restricted to essential information and were treated to guarantee the respect of the privacy of the involved patients, as specifically stated by Italian Law D. Lgs. n.196 of 2003 about personal data protection. Written informed consent was not required, as this study was retrospective and had no patient-identifiable information.

### 4.4. Statistical Analysis

The data are presented as numbers and percentages for categorical variables for statistical analyses. Continuous variables are expressed as mean ± standard deviation (SD), if normally distributed, or as median and interquartile range (IQR), if normality could not be accepted. The data distribution was evaluated by the Shapiro–Wilk test. Comparisons between groups were made with the Mann–Whitney test as appropriate (i.e., CRP or PCT values between infants with fever versus infants without, and CRP or PCT values between infants with positive blood culture versus infants with negative blood cultures).

A *p*-value < 0.05 was considered statistically significant. The data were analyzed with the MedCalc Software package for Windows, release 12.7 (MedCalc Software version 22, Ostend, Belgium).

## 5. Conclusions

We believe that blood cultures should always be performed when fever persists in neonates and young infants admitted because of bronchiolitis, considering that the sepsis risk is not negligible in this age category. Conversely, we identified no meningitis cases in our cohort, and therefore, our proposed algorithm is to wait for the respiratory panel results before decision-making for a lumbar puncture. Indeed, further studies are needed to understand when low-risk febrile infants with bronchiolitis really require a lumbar puncture.

## Figures and Tables

**Table 1:** Identified microorganisms causing bronchiolitis.

	Patients (n = 242)
Respiratory syncytial virus	198 (81.8%)
Rhinovirus	57 (23.6%)
Metapneumovirus	15 (6.2%)
Parainfluenza virus	12 (5.0%)
Coronavirus not SARS-CoV-2	11 (4.5%)
Influenza virus	8 (3.3%)
Adenovirus	3 (1.2%)
Enterovirus	3 (1.2%)
SARS-CoV-2	2 (0.8%)
Not found	2 (0.8%)

**Table 2:** Infection markers in infants with fever and those without.

	Infants with Fever (*n* = 44)	Infants without Fever (*n* = 198)	*p*-Value
CRP (mg/dL)	1.43 (0.58–2.79)	0.35 (0.09–1.10)	0.003
PCT (ng/mL)	0.23 (0.19–0.38)	0.15 (0.11–0.33)	0.337
Leukocytes/mm^3^	9450 (7560–12,005)	10,190 (8145–12,420)	0.136

*PCT was measured in 27 infants with fever and 108 infants without*.

**Table 3:** Infection markers in infants with positive BCs.

Pathogen	CRP Value (mg/dL)	PCT Value (ng/mL)
*1 Streptococcus pneumoniae*	5.18	90.5
*1 Escherichia coli*	3.84	Not available
*1 Pseudomonas aeruginosa*	1.63	Not available
*1 Staphylococcus aureus*	0.38	Not available
*1 Enterococcus faecium*	0.26	0.14

*The infant with Staphylococcus hominis (considered a contaminant) had a CRP value of 1.23 mg/dL and a PCT value of 2.4 ng/mL and was included among the infants with negative BCs*.

**Table 4:** Infection markers in infants with positive BCs and infants with negative BCs.

	Infants with Positive BCs (*n* = 5)	Infants with Negative BCs (*n* = 61)	*p*-Value
CRP (mg/dL)	1.63 (0.38–3.84)	1.43 (0.43–3.47)	0.373
Leukocytes/mm^3^	8110 (7900–11,070)	10,395 (7768–12,790)	0.304

*PCT values could not be compared because only two contrasting values were available for infants with positive BCs*.

**Table 5:** Infection markers in infants without positive BCs results.

CRP Value (mg/dL)	Patients (*n* = 237)	PCT Value (ng/mL)	Patients (*n* = 133)
>5.0	20	>5.0	6
2.5–4.9	20	2.5–4.9	3
1.0–2.4	39	1.0–2.4	2
<1.0	158	<1.0	122

## Data Availability

All data generated or analyzed during this study are included in this published article. Further inquiries can be directed to the corresponding author.
